# Investigation of a Cluster of Immunization Stress-Related Reactions after Coronavirus Disease 2019 (COVID-19) Vaccination, Thailand, 2021

**DOI:** 10.3390/vaccines10030441

**Published:** 2022-03-14

**Authors:** Chayanit Mahasing, Oiythip Yasopa, Chalo Sansilapin, Thanit Rattanathumsakul, Panithee Thammawijaya, Rapeepong Suphanchaimat, Pawinee Doung-ngern

**Affiliations:** 1 Division of Epidemiology, Department of Disease Control, Ministry of Public Health, Nonthaburi 11000, Thailand; o.thippp@gmail.com (O.Y.); chalosansilapin@gmail.com (C.S.); nigagape@hotmail.com (T.R.); viewfetp@gmail.com (P.T.); rapeepong@ihpp.thaigov.net (R.S.); pawind@gmail.com (P.D.-n.); 2 International Health Policy Program, Ministry of Public Health, Nonthaburi 11000, Thailand

**Keywords:** adverse event following immunization, immunization stress-related reaction, COVID-19 vaccination, CoronaVac

## Abstract

On 28 April 2021, the investigation team of the Department of Disease Control, Ministry of Public Health, was notified of a cluster of people developing neurological symptoms following COVID-19 vaccination in a province in eastern Thailand. We conducted an investigation from 29 April to 20 May 2021 to confirm the outbreak, describe the epidemiological characteristics and identify possible risk factors. A matched case-control study was conducted. Matching factors were gender and vaccination site. A confirmed case was a person receiving COVID-19 vaccination in the province and developed at least one neurological symptom between 21 April and 20 May 2021. The rapid assessment of the vaccination cold chain system was carried out. We found a total of 36 cases out of 3920 vaccinees (attack rate = 0.92%), all cases were recovered and classified as an immunization stress-related reaction (ISRR) by the National AEFI Expert Committee. An analytic study found that menstruation was significantly associated with ISRR (AOR = 6.84 [95%CI = 1.09–42.91]). The environmental survey suggested that the cold chain system was properly managed. Further studies on other precipitating causes of ISRR should be performed. In terms of recommendation, health providers should pay greater attention to women menstruating during and after COVID-19 immunization.

## 1. Introduction

The term immunization stress-related reaction (ISRR) is used to describe a range of signs and symptoms that are related to “anxiety” around immunization and are not necessarily directly related to the vaccine product, a defect in the quality of the vaccine, or an error of the immunization program. Stress responses are complex, involving a combination of physiological factors, psychological strengths and the social context. The reasons for which an individual presents stress can be explained by the biopsychosocial model. ISRR can manifest as an acute stress response, vasovagal reactions, or a dissociative neurological symptom reaction (DNSR) [1].

DNSR is characterized by neurological symptoms with no structural findings, otherwise known as functional neurological symptoms. It may present as weakness or paralysis, tingling sensations in the muscles, speech difficulties, abnormal movements or limb posturing, gait irregularities, and non-epileptic seizures with no apparent physiological basis [1,2]. The onset may take hours to days after immunization [1]. Though DNSR has not been well documented or reported in individuals following immunization, there are reports of clusters of these reactions in multiple people in close proximity in tetanus, tetanus-diphtheria, hepatitis B, oral cholera, human papillomavirus and influenza A (H1N1) vaccination [2,3]. DNSRs appear to be more common in females and adolescents [1]. These anxiety-related adverse events following immunization (AEFI) clusters can be disruptive to vaccination programs, reducing public trust in immunizations and impacting vaccination coverage [3].

Given the Coronavirus Disease 2019 (COVID-19) pandemic, the vaccination programs were expedited in almost all countries. The report of severe adverse effects more or less caused worry about vaccine safety and created a reluctance in undertaking immunization in the wider public [4]. It is critical to determine whether COVID vaccines cause neurologic disorders (such as vaccine-related demyelinating diseases, fever-induced seizure, and other possible deficits). Current evidence indicates a minor risk of acute neurological disorders after COVID-19 immunization [5].

The COVID-19 vaccines in Thailand were first administered in March 2021. The healthcare workers, the elderly and people with medical conditions were considered the priority. CoronaVac, the inactivated vaccine platform, was the only vaccine available in Thailand between March and May 2021; then AstraZeneca vaccines—the viral vector vaccine—were supplied in early June 2021. The inactivated vaccine is one of the most traditional platforms which have been previously proved for safety (such as in the case of vaccines against the influenza virus) [5].

Between 1 March and 30 April 2021, fifty-seven neurological events following COVID-19 vaccination were reported in Thailand. The cases were reported to the Adverse Event Following Immunization (AEFI) surveillance database of the Division of Epidemiology (DOE), the Ministry of Public Health (MOPH) [6]. The cases received CoronaVac of different lot numbers which were distributed throughout the entire country. The first remarkable report was when six healthcare workers (HCWs) in a province developed neurological symptoms after vaccination. They were provisionally diagnosed with ischemic stroke. As a result, five out of six received thrombolytic drugs. All of them were female and two were during their menstrual period [7]. This issue was broadcasted and rapidly shared via social media from 20 April 2021 and it greatly raised concern about the immunization program amongst the general public at that time.

On 28 April 2021, the DOE was notified by a local health officer that there was a cluster of AEFI after COVID-19 vaccination in Hospital E, Province S—a key border province in the eastern region. Ten developed neurological symptoms after receiving the COVID-19 vaccination on 21 April 2021. The investigation team conducted an investigation of this cluster from 29 April to 20 May 2021 to confirm the diagnosis, describe the epidemiological characteristics of the outbreak, and identify possible risk factors. Findings from this investigation can help provide new insights into the adverse events following COVID-19 immunization and useful recommendations for appropriate control measures to improve the COVID-19 immunization program.

## 2. Material and Methods

The investigation consisted of two steps: (i) descriptive epidemiological investigation, and (ii) analytic study.

### 2.1. Descriptive Epidemiological Investigation

To confirm the diagnosis and describe epidemiological characteristics of the event, we reviewed medical records and neuroimaging of cases, interviewed cases and physicians, and reviewed the National AEFI Expert Committee Meeting causality assessment reports regarding the cases’ symptoms and diagnosis. Active case finding (ACF) was performed using the Provincial Health Office (PHO) database and Mo Prom database (the national mobile application for monitoring the vaccination program), and the DOE’s AEFI surveillance database of COVID-19 vaccination in Thailand. A confirmed case was an individual who got vaccinated with the COVID-19 vaccine on 21 April 2021 in Province S and developed at least one of the following neurological symptoms: paresthesia, weakness, dysarthria or seizure between 21 April to 20 May 2021; and was both verbally and physically examined for signs and symptoms by a physician. A probable case was those who were only verbally examined for signs and symptoms by a physician. The confirmed cases were followed up 2 times, including 1 month after the first dose to evaluate the clinical course and 4 months after the first dose to evaluate the signs and symptoms after their preferred second dose of COVID-19 vaccine, as there were alternative vaccines at that time.

### 2.2. Analytical Study

#### Study Design

A matched case-control study was performed, and cases and controls were matched by gender and vaccination site. We defined a case as a person who met the confirmed case definition from the ACF as they were examined by the physicians. Those who were uncontactable were excluded from this study. A control was defined as a person who received the COVID-19 vaccine on 21 April 2021 in Province S and did not have any of the neurological symptoms (paresthesia, weakness, dysarthria or seizure) from 21 April to 20 May 2021. The controls were recruited from Mo Prom database. We matched using a control-to-case ratio of 4 to 1.

The sample size estimation followed a formula for matched case-control studies, as suggested by Dupon [8]. In the actual fieldwork, we could identify a total of 18 cases and 72 controls. As the participant toll (*n* = 90) was under our managerial capacity, we recruited all of them in the study without further sampling. This number corresponds with the minimum sample size that was able to detect the expected odds ratio equal to 5.5, given the probability of exposure (having a menstrual period) among cases as 40%, the correlation coefficient for the exposure variable and matching variable was 0.2, a power of 80% and Type I error probability of 5%.

### 2.3. Data Analysis

Both descriptive statistics and inferential statistics were applied using STATA version 14^®^ [9]. For descriptive statistics, frequency and percentage were used to explain demographic data and the possible risk factors. For inferential statistics, conditional logistic regression analysis was performed to evaluate the association of various risk factors with the risk of ISRR, as displayed in the forms of odds ratios (OR) with a 95% confidence interval (95%CI). The inferential statistics were divided into two steps: (i) univariable analysis and (ii) multivariable analysis. Variables with a *p*-value < 0.2 in the univariable analysis or probable risk factors from previous studies were included in the multivariable analysis. According to the small number of cases in the study, we limited the number of exposure variables in the multivariable analysis to three variables [10]. In the univariable analysis, there were four variables that had a *p*-value < 0.2. More detailed findings of the univariable and multivariable analyses are presented later in the results section. A *p*-value < 0.05 was considered statistically significant. Other possible combinations of the exposure variables are presented in Supplement Table S1.

### 2.4. Environmental Study

The environmental survey of the cold chain system of the vaccines was performed in four hospitals where the confirmed cases were identified. The investigation team explored the process of vaccine transfer and reviewed the temperature record during transportation from PHO, during preservation in the hospitals and during the vaccination process. We also conducted a walk-through survey of the vaccination sites, including the screening station, vaccinated station and observation station to explore the temperature and capacity of the vaccination site.

## 3. Results

### 3.1. Descriptive Epidemiological Investigation

On 21 April 2021, the same lot of CoronaVac vaccines were distributed to 12 different vaccination sites and were administered to 3920 people in Province S. Most of them were female (62%) and their median age was 38 years (interquartile range [IQR], 29–47). A total of these vaccines were administered in one day and they were all the first dose of COVID-19 vaccination. A total of 36 cases of neurological AEFI (21 confirmed cases and 15 probable cases) were observed from 21 April to 20 May 2021, corresponding to an overall attack rate of 0.92%. All cases received a similar vaccine lot number with 24 different serial numbers. The cases were distributed in six vaccination sites with the specific attack rate, as shown in Table 1. The median age was 31 years (IQR, 26–38) and the majority of cases (29; 81%) were female. Twelve (33%) cases reported having existing medical conditions, such as allergic rhinitis (8%), dyslipidemia (5%), hypertension (3%) and asthma (3%). Of the 36 cases, 30 (83%) were HCWs. Among the HCWs, 3 (10%) were physicians, 11 (37%) were nurses, 5 (17%) were public health officers, and 3 (10%) were dentistry officers. The cases were interviewed for the possible risk factors. Amongst female cases (29), 7 (24%) were on their menstrual period and 6 (21%) used contraceptive drugs for the past 1 month before vaccination. Clinical symptoms of illness included paresthesia (100%), headache (28%), dizziness (22%), weakness (22%), chest discomfort (19%), diarrhea (17%) and dysarthria (8%), respectively. The median duration from vaccination to onset was 18 h. The minimum onset was 5 min, and the maximum onset was 6 days. The majority of cases (44%) had symptoms during 30 min to 24 h after vaccination. Weakness symptoms always presented during the first 24 h. (Table 2).

Causality assessment was base performed by the National AEFI Expert Committee; all confirmed cases (21) were classified as ISRR by the Committee. We followed the symptoms of the confirmed cases, and all of them had fully recovered within 30 days. Eighteen cases (86%) were contactable. Of these 18 cases, 15 (83%) rejected to receive the second dose on the appointment date. On second follow-up, seventeen cases (81%) remained contactable, 13 (77%) had received the second dose of vaccination. The second dose vaccines included AstraZeneca (6), Comirnaty (Pfizer) (3) and CoronaVac (4), as, during that time, the Thai guidelines allowed a mixed vaccine regimen. Two cases reported paresthesia after the second dose (one after CoronaVac and the other one after Comirnaty). Both fully recovered within a week.

### 3.2. Analytical Study

In the univariable analysis by conditional logistic regression, we identified four potential risk factors, namely, menstruating (OR = 8.80 [95% CI = 1.73–44.85]), being a HCW (OR = 6.21 [95% CI = 1.68–22.93]), aged ≤ 30 years (OR = 3.56 [95% CI = 1.25–10.14]) and having a comorbidity related to allergic respiratory diseases (allergic rhinitis and asthma) (OR = 2.57 [95% CI = 0.67–9.83]). The most profound risk factor was menstruation. Among seven confirmed cases whom we could contact, six (43%) were on the period, whereas there were only 5 (9%) in controls. Compared with those who were not on the period, participants who were on the period faced greater odds of being a case by about 8.8 times. HCWs constituted about 78% of cases and 38% of controls. The odds of being a case in HCWs were 6.21 larger than the odds in non-HCW participants in the univariable analysis (Table 3 and Table 4). In the multivariable analysis, we divided the analysis into two models: first by including being HCWs, age group and menstruating as independent variables (model 1), and second, by using being HCWs, history of underlying allergic respiratory diseases and menstruating as independent variables (model 2), since we limited the number of independent variables to three variables (more details in the Materials and Methods section). The first model illustrated that by controlling for HCWs and age group, the people who were menstruating had 6.84 times greater odds of being a case compared with those who were not menstruating (AOR = 6.84 [95% CI 1.09–42.91]). The large AOR in menstrual variables, also presented in model 2, showed that by controlling for HCWs and underlying allergic respiratory diseases, people who were menstruating presented 8.55 times larger odds of being a case compared with those who were not menstruating (AOR = 8.55 [95% CI 1.36–53.91]). However, being an HCW did not show a significant difference after controlling for menstruation and age group (AOR = 3.63 [95% CI 0.93–14.24]) with *p*-value = 0.64 (model 1) or after adjusting for menstruation and allergic respiratory diseases (AOR = 4.03 [95% CI 0.97–16.81]) with *p*-value = 0.56 (model 2). Statistical significance was neither found in age group nor allergic diseases in both models (Table 5).

### 3.3. Environmental Study

The CoronaVac vaccines were transported from the Government Pharmaceutical Organization (GPO) to the store in Hospital E. They were distributed to all vaccination sites in Province S on 20 April 2021 and stored in the pharmacy sectors in each hospital before being administered on 21 April 2021. During transportation, the temperature was controlled at 2–8 degrees Celsius and was monitored by a data locker every 5 min. The temperature in the storage was controlled by the pharmacists if it was out of range. There was no cold chain breakdown reported in the four hospitals we surveyed. During the vaccination, the vaccines were contained in the ice boxes with temperature monitoring. There was no vaccine package damage. The process from screening to vaccination was approximately 30 min. All vaccinated persons were observed for any adverse events for 30 min at the vaccination sites. The total vaccination process lasted nearly an hour. The vaccination sites had enough space for physical distancing. The vaccinated stations were separated from the observation station. The airflow was well ventilated.

## 4. Discussion

The study confirms the presence and magnitude of the outbreak of neurological AEFI in a province, in Thailand. An anxiety-related reaction after immunization was likely to be the main cause of this event. The widespread news regarding clusters of neurological deficits during that time might influence public concern about vaccine safety [7,11]. Unlike structural neurogenic disorders, all cases had transient symptoms and fully recovered after follow-up. Although all cases received the same lot and type of vaccines in the first dose, some of them had similar symptoms when they received different lots and types of vaccine for the second dose (one with a different lot of CoronaVac and one with Comirnaty). The findings in this event corresponded with the biopsychosocial concept of immunization stress-related responses that the specific vaccine was not included in the physiological factor [1]. This phenomenon was also observed elsewhere outside Thailand. A systematic review by Loharikar et al. also suggested that anxiety-related AEFI could occur in different lots and types of vaccines [3]. Moreover, the AEFI surveillance data of the DOE also illustrated that these symptoms could occur in different vaccination services across the country [6]. In addition, the cold chain systems and environment of the vaccination service were properly managed. Hence, the reactions due to the vaccine product, vaccine quality defect, and immunization error were less likely in this event.

All cases were classified as ISRR according to the national AEFI experts. Cases occurred mainly in women of young age. Most had symptoms within 24 h and recovered completely within a week. The symptoms were positively correlated with DNSR, including weakness and tingling sensations in the muscles and it usually happens hours to days after immunization. Prior literature also reports that DNSR is more common among females and people of early age [1,2,3]. Furthermore, DNSR clusters usually occurred in a new vaccine introduction, including a novel vaccine, a new age group, or a new setting for vaccination [1]. As in this event, CoronaVac vaccines were the first (and a new) COVID-19 vaccine introduced in Thailand at that time, and all cases received these vaccines as their first dose of COVID-19 vaccination. The clusters of anxiety-related reactions after the COVID-19 vaccination—with the Janssen COVID-19 vaccine—also occurred in the United States and were after five weeks’ use of the Janssen COVID-19 vaccine. Most cases occurred in women (61%) and young age (median age 36 years). The most common symptoms were light-headedness. However, some cases reported abnormal neurological symptoms; seizure-like activity [12].

Being in their menstruation period showed a statistically significant association with the development of ISRR, both when adjusted with being HCW and age group (model 1) and with being HCW and with a history of underlying allergic respiratory diseases (model 2). Although prior evidence did not point to a clear association between menstruation and neurological symptoms following vaccination, there was a report that female stress responses were related to stress sensitivity through increased awareness and processing of social cues in a stressful context, with the menstrual cycle phase being a critical factor [13]. In addition, about half of reproductive-age women were affected by perimenstrual distress, which could present in various forms, such as irritability, anxiety, fatigue or depression around the menstrual process [14,15]. In this regard, contraceptive drug use, which is known as a hormonal factor that increased the risk of venous and arterial thromboembolism [16,17], did not demonstrate a significant relationship with ISRR. Thus, menstruation was less likely associated with ISRR in terms of biological aspects as the hormonal factor. On the other hand, psycho-social factors might play a pivotal role in this context compared with biological determinants. Similar to the Centers for Disease Control and Prevention (CDC) recommendation, the Thai national AEFI experts recommended that menstruation is not a contraindication for COVID-19 vaccination. However, the committee recommended that management at the vaccination site to calm down the vaccinees is crucial [18,19].

Our investigation showed that being HCW appears to be associated with ISRR, although the strength of the association in the multivariable model did not show a statistical significance. A possible reason was HCWs were more able to recognize and be aware of any abnormal symptoms and could easily access healthcare services due to familiarity with the healthcare system than the general population. In addition, the COVID-19 situation might cause more anxiety in frontline health personnel (particularly nurses) than the non-HCW population [20]. Information from the World Health Organization (WHO) shows that ISRR is more common in specific occupational groups, including healthcare personnel, students and military reservists [1,3].

From a public health perspective, the AEFI cluster, to some extent, brought about vaccination hesitancy nationwide [7]. Although the MOPH in collaboration with the Neurological Society of Thailand, published a guideline detailing information about the disease and vaccine safety, people still reject the second dose of CoronaVac [21]. An earlier report from WHO suggested that widespread misinformation about AEFI over the past few decades caused mistrust and vaccination hesitancy worldwide, and as such, the situation was ranked as one of the top ten threats to the global health arena in 2019 [22]. Therefore, it is crucial to educate the public by way of clear communication that it was unrelated to the vaccine product, immunization program or procedural error. Moreover, health care providers should differentiate ISRR from other conditions, including neurological diagnoses. If ISRR is confirmed, medication and hospitalization should be avoided, as they may aggravate the situation and result in additional cases [1].

This study encountered some limitations. Firstly, ISRR clusters are complex phenomena that involve not only medical determinants but also societal factors which are difficult to observe during field investigation. Secondly, the timing of the interview with the control group occurred about a month after vaccination. Therefore, memory bias could cause inaccuracy of the data collected, such as the date of the menstrual period. Lastly, as this study was part of the actual investigation of the DOE, we could not determine the number of cases from the outset. For this study, the volume of cases was quite small (as we included only confirmed cases in the analytic study instead of probable and confirmed cases combined in order to avoid a misclassification bias), causing a restriction of the number of factors to be included in the multivariable analysis due to events-per-variable criteria. This meant we could not account for many confounding factors at the same time in the multivariable conditional logistic regression.

## 5. Conclusions

We confirmed the ISRR cluster in Province S following CoronaVac vaccination. The majority of cases were found amongst females, healthcare workers and people of young age. In terms of recommendations, it is crucial for the vaccination sites to identify individuals with predisposing risk factors of ISRR and to implement measures to reduce stresses of the vaccinees, such as modifying injecting position, introducing pain reduction procedures, and creating a friendly environment for vaccination. Being in the menstrual period was probably a significant predisposing factor, thus healthcare personnel responsible for vaccine administration should be aware of ISRR, particularly amongst women menstruating. However, further studies on the psycho-social factors that may play an important role in women menstruating and ISRR after COVID-19 immunization are recommended. For hospitals, it is vital to distinguish ISRR from other conditions, including neurologic diagnoses. The management of an ISRR necessitates a multidisciplinary approach. For the public health offices, assuring the public through clear communication is needed and the officers should be vigilant over signs and symptoms which may signal ISRR as part of the national AEFI surveillance.

## Figures and Tables

**Table 1:** Distribution of CoronaVac vaccine and number of cases by vaccination sites.

Vaccination Sites (Hospital)	Number of Doses	Confirmed Cases	Probable Cases	Total Cases	Attack Rate (%)
Hospital A	200	0	4	4	2.00
Hospital B	320	4	2	6	1.88
Hospital C	400	5	2	7	1.75
Hospital D	200	2	0	2	1.00
Hospital E	1600	10	6	16	1.00
Hospital F	520	0	1	1	0.19
Hospital G	160	0	0	0	0.00
Hospital H	80	0	0	0	0.00
Hospital I	40	0	0	0	0.00
Hospital J	120	0	0	0	0.00
Hospital K	200	0	0	0	0.00
Hospital L	80	0	0	0	0.00
Total	3920	21	15	36	0.92

**Table 2:** Demographic data and clinical features for cases.

Features	Categories	Cases (*n* = 36)	% of Total Cases
Age group (years)	20–29	15	42
	30–39	13	36
	40–49	4	14
	50–59	3	8
Gender	Male	7	19
	Female	29	81
Existing medical conditions	Yes	12	33
	No	24	67
Medical conditions (*n* = 12)	Allergic respiratory disease	4	33
	Dyslipidemia	2	17
	Arrhythmia	2	17
	Bipolar	1	8
	Endometriosis	1	8
	Hypertension	1	8
	Hyperthyroid	1	8
Medical service	Inpatient	17	47
	Outpatient	4	11
	Self-remission	15	42
Career	HCW	30	83
	Non-HCW	4	11
	No info	2	6
Health care professionals (*n* = 30)	Physician	3	10
	Nurse	11	37
	Public health officer	5	17
	Dentistry officer	3	10
	Pharmacy officer	2	7
	Supplies officer	2	7
	Administrator	1	3
	IT officer	1	3
	Porter	1	3
	Radiologist	1	3
Clinical manifestation *	Paresthesia	36	100
	Headache	10	28
	Dizziness	8	22
	Weakness	8	22
	Chest discomfort	7	19
	Diarrhea	6	17
	Dysarthria	3	8
Duration of onset	Within 30 min	5	14
	30 min to 24 h	16	44
	1–3 days	9	25
	3–6 days	4	11
	No info	2	6
Recovery period (*n* = 18)	Within 7 days	6	33
	7–14 days	4	22
	14–21 days	6	33
	21–30 days	2	11
History of migraine	Yes	29	81
	No	4	11
	No info	3	8
History of drug allergy	Yes	3	8
	No	32	89
	No info	1	3
Menstrual status during vaccination, females (*n* = 29)	Yes	7	24
	No	19	66
	No info	3	10
Contraceptive drug use in previous month, females (*n* = 29)	Yes	6	21
	No	21	72
	No info	2	7
Median hours of sleep the day before vaccination (P25–P75)	-	-	7 (6–8)

Note: HCW = health care worker; * Percent in row.

**Table 3:** Characteristics of ISRR cases and matched controls ^§^.

	Cases (%)	Controls (%)
	*n* = 18	*n* = 72
Demographic factors (*n* = 90)		
Age group		
Age ≤ 30	10 (56)	17 (24)
Age > 30	8 (44)	55 (76)
Occupational group		
Non HCW	4 (22)	45 (62)
HCW	14 (78)	27 (38)
Medical factors (*n* = 90)		
Non-communicable diseases ^#^		
No	16 (89)	65 (90)
Yes	2 (11)	7 (10)
Allergic respiratory diseases		
No	14 (78)	65 (90)
Yes	4 (22)	7 (10)
History drug allergy		
No	17 (94)	67 (93)
Yes	1 (6)	5 (7)
History of migraine		
No	16 (89)	63 (88)
Yes	2 (11)	2 (12)
History of ergotamine use in the last month		
No	18 (100)	71 (99)
Yes	0 (0)	1 (1)
Hormonal factor (*n* = 70)		
History of contraceptive drug use in last 1 month		
No	14 (100)	46 (82)
Yes	0 (0)	10 (18)
Being in menstrual period during vaccination		
No	8 (57)	51 (91)
Yes	6 (43)	5 (9)
Behavioral factors (*n* = 90)		
Caffeine drink within a day before vaccination		
No	12 (67)	37 (51)
Yes	6 (33)	35 (49)
Energy drink within a day before vaccination		
No	18 (100)	70 (97)
Yes	0 (0)	2 (3)
AEFI news recognition		
No	4 (22)	11 (15)
Yes	14 (78)	61 (85)
Sleeping hours		
Median (P25–P75)	6.5 (6–7.5)	6.5 (6–7.5)

Note: ISRR: immunization stress-related response; HCW: health care worker; ^#^: Including hypertension and dyslipidemia. ^§^ There were 14 women in the case group and 56 women in the control group.

**Table 4:** Univariable conditional logistic regression analysis to estimate the odds of ISRR.

	Unadjusted OR	95% CI	*p*-Value
Demographic factors (*n* = 90)			
Age group * (ref = Age > 30)			
Age ≤ 30	3.56	1.25–10.14	0.017
Occupational group * (ref = Non HCW)			
HCW	6.21	1.68–22.93	0.006
Medical factors (*n* = 90)			
Non-communicable diseases ^#^ (ref = No)			
Yes	1.15	0.23–5.83	0.864
Allergic respiratory diseases * (ref = No)			
Yes	2.57	0.67–9.83	0.168
History drug allergy (ref = No)			
Yes	0.79	0.08–7.28	0.832
History of migraine (ref = No)			
Yes	0.87	0.16–4.66	0.868
History of ergotamine use in the last month (ref = No)			
Yes	-	-	-
Hormonal factor (*n* = 70)			
History of contraceptive drug use in last 1 month (ref = No)			
Yes	-	-	-
Being in menstrual period during vaccination * (ref = No)			
Yes	8.8	1.73–44.85	0.009
Behavioral factors (*n* = 90)			
Caffeine drink within a day before vaccination (ref = No)			
Yes	0.5	0.16–1.62	0.249
Energy drink within a day before vaccination (ref = No)			
Yes	-	-	-
AEFI news recognition (ref = No)			
Yes	0.63	0.18–2.27	0.482
Sleeping hours			
One hour increment in sleeping duration	1.07	0.77–1.49	0.67

Note: ISRR: immunization stress-related response; HCW: health care worker; OR: odds ratio; 95% CI: 95% confidence interval; ref: reference; ^#^ Including hypertension and dyslipidemia; * Denoting variables that were later used in the multivariate analysis.

**Table 5:** Multivariable conditional logistic regression analysis to estimate the odds of ISRR.

	Model 1			Model 2		
	AOR	95% CI	*p*-Value	AOR	95% CI	*p*-Value
Age group (ref = Age > 30)						
Age ≤ 30	1.69	0.44–6.52	0.45	-	-	-
Occupational group (ref = non-HCW)						
HCW	3.63	0.93–14.24	0.064	4.03	0.97–16.81	0.056
Allergic respiratory disease group (ref = no)						
Yes	-	-	-	4.03	0.54–30.10	0.175
Menstrual period (ref = no)						
Yes	6.84	1.09–42.91	0.04	8.55	1.36–53.91	0.022

Note: ISRR: immunization stress-related response; HCW: health care worker; AOR: adjusted odds ratio; 95% CI: 95% confidence interval; ref: reference.

## Data Availability

Not applicable.

## Supplementary Materials

The following supporting information can be downloaded at: https://www.mdpi.com/article/10.3390/vaccines10030441/s1, Table S1: Multivariable conditional logistic regression analysis to estimate the odds of ISRR (model 3 and model 4).
