# Supplementary material for: Investigation of a Cluster of Immunization Stress-Related Reactions after Coronavirus Disease 2019 (COVID-19) Vaccination, Thailand, 2021
Source: Vaccines (Basel). 2022 Mar 14;10(3):441. doi: 10.3390/vaccines10030441 (PMC8954559; doi:10.3390/vaccines10030441)
Supplement: Supplementary file 1 [file vaccines-10-00441-s001.zip › vaccines-1572333-supplementary.pdf]

vaccines

Supplementary Materials

Table S1. Multivariable conditional logistic regression analysis to estimate the odds of ISRR.

	Model 3			Model 4		
	**AOR**	**95%CI**	***p*-Value**	**AOR**	**95%CI**	***p*-Value**
Age group (ref = Age > 30)						
Age ≤ 30	2.31	0.62–8.54	0.211	3.07	0.94–10.02	0.062
Occupational group (ref = non-HCW)						
HCW	-	-	-	5.33	1.33–21.36	0.018
Allergic respiratory disease group (ref = no)						
Yes	4.11	0.73–23.12	0.109	3.22	0.64–16.07	0.155
Menstrual period (ref = no)						
Yes	7.63	1.45–40.24	0.017	-	-	-
